# How do Ontario family medicine residents perform on global health competencies? A multi-institutional survey

**Published:** 2013-09-30

**Authors:** Mirella Veras, Kevin Pottie, Tim Ramsay, Vivian Welch, Peter Tugwell

**Affiliations:** 1 Center for Global Health, University of Ottawa, Ontario, Canada; 2 Department of Family Medicine, University of Ottawa, Ontario, Canada; 3 Clinical Epidemiology Program, Ottawa Hospital Research Institute, Ontario, Canada; 4 Bruyere research institute, University of Ottawa, Ontario, Canada; 5 Department of Medicine, Faculty of Medicine, University of Ottawa, Ontario, Canada

## Abstract

**Background:**

There is an increased interest in global health among medical students, family medicine residents, and medical educators. This paper is based on research to assess confidence in knowledge and skills in global health in family medicine residents in five universities across Ontario.

**Methods:**

A web-based survey was sent to 166 first-year family medicine residents from five universities within Ontario. Descriptive statistics were used to analyze residents’ confidence in their knowledge and skills in global health. The strength of association between each of the self-perceived knowledge and skills variables was assessed by the Spearman correlation coefficient

**Results:**

The response rate ranged from 29% to 66% across the five universities. Self-perceived knowledge scores revealed that 34.3% of the respondents were very confident, 51.9% were somewhat confident, and 13.8% were not at all confident about their global health knowledge. Participants’ confidence scores were lower in relation to knowledge of access to health care for low income nations (44.3%), and were better on their global health skills related to working in a team (70.9%) and listening actively to patients’ concerns (64.6%).

**Conclusions:**

The global health competency scale has identified key areas of strengths and weaknesses of family medicine programs in global health education. This can be used to evaluate and analyze progress over time.

## Introduction

There is a marked increase in interest in global health among medical students, family medicine residents and medical educators;^1^,^2^ they are demanding more emphasis on global health teaching and international opportunities in medical school.^3^,^4^ In these times of globalization,^5^ it is important that family physicians feel competent in contributing meaningfully to reducing inequalities, both in their own practice and in the global context with determinants of health such as socioeconomic, environmental and political factors. Hence, global health training should be part of the residency program curriculum and be adapted to the requirements of each specialty.^6^ Global health training may further stimulate students to develop a commitment to community health as well as provide an opportunity to work with socially disadvantaged populations. Additional benefits of global health training include an opportunity to learn or improve a foreign language.^7^

A key component of global health are the social determinants of health identified by the World Health Organization*.* In Canada, health disparities are related mainly to ethnic background, gender, education level, income, geographic location, and other characteristics that can bring disadvantages in accessing health care for some groups compared to others.^8^ Family physicians have a crucial role to play in providing effective primary health care for disadvantaged populations, and primary care has been shown to be an effective strategy to promote health equity in both high-income and low and middle-income countries.^9^

The quality of education for family medicine residents plays an important role in the effectiveness of primary care.^10^ Improving family physicians’ knowledge about global health and health equity may help to reduce health care disparities, address the inequities related to treatment and access to health care for socially disadvantaged populations,^11^ and achieve effective primary care. Canadian health system policies put family medicine central in delivering comprehensive community-based health care. Canada is large, ethnically diverse, has indigenous health inequalities, a universal health care system that covers the entire population, and has primary care as a priority area in health.

Therefore, improving education in global health and health equity for family medicine residents has a huge potential to enhance family medicine residency programs and consequently improve the effectiveness of primary care services for socially disadvantaged populations. This study is part of a larger assessment of global health competency in students in healthcare. This study aims to describe global health competency in family medicine residents in Ontario in order to understand their perceived knowledge and skills in global health as well as learning needs in major global health topics.

## Methods

### Sample

In 2011, a total of 452 family medicine residents in five Ontario universities were invited to participate in the survey. The criteria for inclusion in this study were: being 18 years or older; being in a program from the following participating universities: University of Ottawa, University of Toronto, Queen’s University, Western University and McMaster University; and must be a 1^st^ year resident in a family medicine residency program.

### Design

A cross-sectional study.

### Data collection

Residents who were eligible to participate were identified by the directors or coordinators of their program. They received an electronic e-mail invitation with a web link to access the online survey and consent form. Reminders were sent at two-week intervals. The data were collected using a web-based tool, Survey Monkey (http://www.surveymonkey.com).

### Instrument

The instrument was adapted from: (a) a validated questionnaire used to measure actual and perceived resident physicians’ knowledge of underserved patient populations in the United States and adapted to the Canadian population,^12^ (b) items from a global health competency survey for medical students,^13^ and (c) Canadian Medical Education Directives for Specialists (CanMEDS) competencies.^14^ The instrument was assessed for validity, reliability and pretested in a previous study. The survey consisted of 30 questions subdivided into four categories: 1) Knowledge of global health and health equity (self-assessment); 2) Global health skills for working with patients who have different linguistic, educational, socioeconomic, and cultural backgrounds (self-assessment); 3) Learning needs about global health; and 4) About you – socioeconomic and demographic questions. This survey was previously validated and demonstrated good internal consistency and validity with a Cronbach’s alpha > 0.8.^15^

The responses to the questions were: ‘not at all confident’, ‘somewhat confident’ or ‘very confident’. These were coded as 0 (not at all), 0.5 (somewhat) and 1 (very). Thus, by averaging all respondents’ answers to a given question and multiplying that average by 100, each question could be summarized by a number between 0 and 100, with 0 representing an overall complete lack of confidence and 100 representing a perception of being very confident. This approach to measuring confidence was previously used by Wieland et al.^16^

### Ethical considerations

The study was approved by the University of Ottawa, the Ottawa Hospital research Ethics Board, and the University of Western Ontario.

### Data analysis

The data were analyzed with descriptive statistics using the Statistical Package for the Social Sciences Software (SPSS, version 19). Descriptive statistics were used to analyze residents’ self-perceived knowledge and skills in global health. The strength of association between each of the global health knowledge and skills variables was assessed with the Spearman correlation coefficient.

## Results

### Demographic characteristics

Surveys were completed by 166 residents. The overall response rate ranged from 29% to 66.35%. Respondents were predominantly female (68.7%), median age was 29 years, white background (58.4%), raised by parents with family income of $80,000 or more per year (44.6%), and able to speak only one language (42.8%) (Table 1).

### Self-perceived knowledge

Self-perceived knowledge in global health topics revealed that 34.3% of the respondents were very confident, 51.9% were somewhat confident, and 13.8% were not at all confident. Regarding all topics, residents’ average score percentages were higher for confidence in their knowledge of the relationship between income and health (79.52%), relationship between socioeconomic position and impact on health (75.30%), and relationship between work and health (70.90%). Their average score percentages were lower for access to healthcare for low income nations (44.24%), mechanisms for why racial and ethnic disparities exist (44.58%), and racial stereotyping and medical decision making (46.39%) (item scale: 0 = not at all confident; 0.5 = somewhat confident; 1 = very confident) (Table 2).

### Self-perceived global health skills related to the CanMEDS framework

Residents had better scores on confidence in their global health skills in working in a team (70.93), listening actively to patients concerns (64.55), and clinical competency for working with patients with different backgrounds (64.31). The three skills with lower scores were: helping patients from different backgrounds achieve realistic goals (38.16); keeping up to date in global health (42.62), and active in global health skills (42.92) (item scale: 1 = Strongly disagree; 0.75 = Disagree; 0.50 = Neutral ; 0.25 = Agree; 0 = Strongly agree). For positive questions (1 = Strongly agree; 0.75 = Agree; 0.50 = Neutral ; 0.25 = Disagree; 0 = Strongly disagree). (Table 3).

### Correlation between self-perceived knowledge of global health and global health skills

As shown in Table 4, several variables from self-perceived knowledge of global health were significantly correlated with global health skills variables. The major positive significant correlations were found for: 1) residents’ knowledge of racial and ethnic disparities and skills in keeping up to date in global health (0.38); 2) knowledge of racial stereotyping and clinical decision making and skills in keeping up to date in global health skills (0.35); 3) knowledge of gender and access to health care and skills in keeping up to date in global health (0.34).

## Discussion

Our results show that family medicine residents’ confidence in their knowledge of global health was generally low. These results were corroborated by the findings of a survey of US residents on actual and perceived knowledge of underserved populations which also showed low self-perceived knowledge (14% of the residents were very confident, 66.4% were somewhat confident, and 19.6% were not at all confident). Ontario family medicine residents scored higher than their US counter parts in the relationship between health and income (Ontario family medicine residents 79.52% versus US family medicine residents 57.4%); access to health care for low income nations (Ontario residents: 44.24% versus US residents % 35.7%), language barriers in the health system (Ontario residents 63.25% versus US residents 61.0%); mechanism whereby socioeconomic position could affect health (Ontario residents 75.30% versus US residents 53.2%); environmental health and socioeconomic position (Ontario residents 58.13% versus US family medicine residents 45.9%); racial stereotyping and medical decision making (Ontario residents 46.39% versus US residents 43.5%). US residents were more confident than the Ontario residents for the following topic: mechanisms for why racial and ethnic disparities exist (45.0% for US residents versus 44.58% for Ontario residents). A global health education survey among German medical students showed that 75.0% of the sample achieved a score which was equal or less than 50% of the maximum achievable score.

The perceived global health competencies encompass elements of the Canadian Medical Education Directives for Specialists (CanMEDS) framework.^14^ The framework identifies the following competencies: medical expert, communicator, collaborator, manager, health advocate, scholar and professional. The self-perceived global health skills were based on these competencies. The family medicine residents in Ontario had a better score in working in a team (which is part of the collaborator competency in the CanMEDS framework), able to understand patients with different background (which is part of the communicator competency in the CanMEDS framework), and listening skills (also part of the communicator competency in the CanMeds framework).

An effective practice in global health settings demands knowledge and skills in all CanMeds competencies. Hence participation in global health activities can help residents learn and develop their skills in global health.^17^ A family physician should be able to communicate effectively with patients from different backgrounds, literacy and language in order to provide effective care.^17^

A biannual survey developed by the SOR Council found that 88% of family medicine programs have some formal global health curriculum and most schools devote less than 10 hours to global health during their training.^18^ This survey includes two residents from each family medicine program across Canada and intends to compare programs nationally.^18^ There is a need to improve global health education across programs in Canada as well as global health skills related to other CanMEDS competencies, such as expert, manager, health advocate, scholar and professional. All these competencies are essential for a family physician to provide effective care to disadvantaged populations locally and internationally.

Although we found small correlations among self-perceived levels of knowledge of global health and self-perceived global health skills, all self-perceived knowledge variables have a correlation with the global health skills, which highlights the importance of the use of this survey to evaluate and track changes in global health skills of family medicine residents.

### Conclusion

This study attempted to describe self-perceived knowledge of global health and global health skills in family medicine residents in Ontario. The findings of this study demonstrate clearly that there is a need to improve global health curricula in family medicine residency programs in Ontario universities. Overall, the self-perceived knowledge of global health score was below 60% for six out of the twelve items. Residents scored below 60% in eight out of eleven global health skills. It is imperative to use and improve this survey tool designed to measure global health competencies. It is also important to evaluate global health knowledge and skills over time to be able to track improvements in the curricula. Therefore, upon review of the survey data, workshops, training and courses can be developed in the short term to address areas of weakness in knowledge and skills in terms of global health competence, considering local and international settings.

## Figures and Tables

**Table 1:** Demographic characteristics of family medicine residents in Ontario, Canada.

Demographic characteristics	*n (%)*
**Sex**	
Male	52 (31.3)
Female	114 (68.7)

**Background**	
White	97 (58.4)
Chinese	19 (11.4)
South Asian	22 (13.3)
Black	4 (2.4)
Latin American	3 (1.8)
West Asian	2 (1.2)
Aboriginal	4 (2.4)
Other	15 (7.7)

**Parents’ family income**	
$20,001 to $30,000	6 (3.6)
$30,001 to $40,000	6 (3.6)
$40,001 to $50,000	9 (5.4)
$50,001 to $60,000	18 (10.8)
$60,001 to $70,000	9 (5.4)
$70,001 to $80,000	17 (10.2)
$80,001 or more	74 (44.6)
Don’t know	27 (16.3)

**Number of languages spoken**	
one language	71 (42.8)
two languages	55 (33.1)
three languages	24 (14.5)
four languages or more	16 (9.6)

**Table 2:** Family medicine residents’ self-perceived global health knowledge

Self-perceived Knowledge	Confidence Score * (%)
Language barrier and adverse impact on health and health care	63.25
Access to health care for low income nations	44.24
Relationship between income and health	79.52
Relationship between work and health	70.91
SEP and impact on health	75.30
Environmental health and socioeconomic position	58.13
Relationship between housing and health status	67.77
SEP and food security confidence	64.46
Health outcome discrepancies among different groups in Canada	54.82
Mechanisms for why racial and ethnic disparities exist	44.58
Racial stereotyping and medical decision making	46.39
Gender and access to health care	54.22

* Average percentage of self-perceived knowledge for each domain varied between 0–1; (item scale: 0 = not at all confident; 0.5 = somewhat confident; 1 = very confident).

**Table 3:** Self-perceived global health skills guided by the CanMEDS framework

Self-perceived Skills	Confidence Score *(%)
Communication	52.86
Listening	64.55
Able to understand patient with different background	54.22
Address team disagreement	55.57
Discuss sensitive issues	51.06
Identify needs	51.66
Helping patients achieve realistic goals	38.16
Working in a team	70.93
Clinical competency	64.31
Keeping up date in Global Health	42.62
Active in Global Health	42.92

* Average percentage of self-perceived global health skills for each domain varied between 0–1.

Item scale for negative questions: 1 = Strongly disagree; 0.75 = Disagree; 0.50 = Neutral ; 0.25 = Agree; 0 = Strongly agree).

Item scale for positive questions: 1 = Strongly agree; 0.75 = Agree; 0.50 = Neutral ; 0.25 = Disagree; 0 = Strongly disagree).

**Table 4:** Spearman correlations among self-perceived knowledge of global health and global health skills.

	Communication	Listening	Able to understand patient background	Address team disagreement	Discuss sensitive issues	Identify needs	Helping patients achieve goals	Working in a team	Clinical competency	Keep up to date in global health	Active in global health
Language barrier	0.124 0.112	0.195* 0.012	0.176* 0.023	0.052 0.503	0.122 0.118	0.158* 0.042	−0.038 0.644	0.094 0.228	0.069 0.380	0.213** 0.006	0.224** 0.004
Access to health care	−0.112 0.152	0.149 0.056	0.198** 0.011	0.083 0.291	0.022 0.779	0.178* 0.022	−0.151 0.064	0.130 0.097	0.014 0.857	0.238* 0.002	0.286** 0.000
Income and health	0.097 0.216	0.148 0.058	0.111 0.154	0.085 0.275	0.183* 0.018	0.037 0.634	−0.014 0.863	0.162* 0.037	0.119 0.128	0.201* 0.009	0.083 0.287
Work and health	0.150 0.055	0.099 0.209	0.136 0.080	0.189* 0.015	−0.004 0.958	0.114 0.147	0.086 0.295	0.186* 0.017	0.086 0.271	0.169* 0.030	0.190* 0.014
SEP and impact on health	0.296* 0.000	0.186* 0.017	0.258** 0.001	0.206* 0.008	0.170* 0.029	0.119 0.126	−0.034 0.679	−0.215** 0.005	0.123 0.114	0.182* 0.019	0.136 0.081
SEP and environmental Health	0.157* 0.043	0.195* 0.012	0.148 0.058	0.134 0.085	0.158* 0.043	0.166* 0.032	−0.048 0.558	0.237* 0.002	0.073 0.349	0.335** 0.000	0.235** 0.002
Housing and health	0.253** 0.001	0.233** 0.003	0.171 0.028	0.220** 0.004	0.065 0.407	0.198* 0.011	0.019 0.816	0.238** 0.002	0.222* 0.004	0.229* 0.003	0.221** 0.004
SEP and food security	0.167* 0.031	0.145 0.062	0.237** 0.002	0.177* 0.022	0.089 0.255	0.162* 0.037	0.021 0.801	0.131 0.093	0.127 0.104	0.277** 0.000	0.256** 0.001
Outcome discrepancies	0.102 0.192	−0.005 0.949	0.264** 0.001	0.136 0.080	−0.058 0.457	0.135 0.083	−0.056 0.494	−0.088 0.257	0.077 0.327	0.291** 0.000	0.270** 0.000
Racial/ethnic disparities	0.188* 0.015	0.167* 0.032	0.181* 0.020	0.050 0.524	0.044 0.571	0.161* 0.038	−0.071 0.386	0.050 0.522	0.028 0.723	0.380** 0.000	0.235** 0.002
Race and clinical decision making	0.186* 0.016	0.071 0.366	0.178** 0.021	0.075 0.338	0.005 0.954	0.103 0.186	0.005 0.951	0.008 0.922	0.167* 0.031	0.348** 0.000	0.189* 0.015
Gender and access to health care	0.239** 0.002	0.183* 0.018	0.177** 0.022	0.146 0.061	0.057 0.465	0.251** 0.001	0.076 0.354	0.117 0.133	0.028 0.721	0.336** 0.000	0.258** 0.001

** Correlation is significant at the 0.01level

* Correlation is significant at the 0.05 level
